# Overview of the Canadian pediatric end-stage renal disease database

**DOI:** 10.1186/1471-2369-11-21

**Published:** 2010-08-26

**Authors:** Susan M Samuel, Marcello A Tonelli, Bethany J Foster, Alberto Nettel-Aguirre, Yingbo Na, Robert Williams, Andrea Soo, Brenda R Hemmelgarn

**Affiliations:** 1 Division of Pediatric Nephrology, Department of Pediatrics, Alberta Children's Hospital, 2888 Shaganappi Trail NW, Calgary, T3B 6A8, Canada; 2 Division of Nephrology, Department of Medicine, University of Alberta, CSB 7-129, Edmonton, T6G 2G3, Canada; 3 Division of Pediatric Nephrology, Department of Pediatrics, Montreal Children's Hospital, 2300 Tupper Street, Montreal, Quebec H3H 1P3, Canada; 4 Department of Pediatrics, Alberta Children's Hospital, 2888 Shaganappi Trail NW, Calgary, T3B 6A8, Canada; 5 Canadian Organ Replacement Register, Canadian Institute for Health Information 495 Richmond Road, Suite 600, Ottawa, K2A 4H6, Canada; 6 Department of Community Health Sciences, Faculty of Medicine, University of Calgary, TRW Building, 3rd Floor, 3280 Hospital Drive NW, Calgary, T2N 4Z6, Canada; 7 Division of Nephrology, Department of Medicine, Foothills Medical Center, 1403-29th St NW, Calgary, T2N 2T9, Canada

## Abstract

**Background:**

Performing clinical research among pediatric end-stage renal disease patients is challenging. Barriers to successful initiation and completion of clinical research projects include small sample sizes and resultant limited statistical power and lack of longitudinal follow-up for hard clinical end-points in most single center studies.

**Description:**

Existing longitudinal organ failure disease registry and administrative health datasets available within a universal access health care system can be used to study outcomes of end-stage renal disease among pediatric patients in Canada. To construct the Canadian Pediatric End-Stage Renal Disease database, registry data were linked to administrative health data through deterministic linkage techniques creating a research database which consists of socio-demographic variables, clinical variables, all-cause hospitalizations, and relevant outcomes (death and renal allograft loss) for this patient population. The research database also allows study of major cardiovascular events using previously validated administrative data definitions.

**Conclusion:**

Organ failure registry linked to health administrative data can be a powerful tool to perform longitudinal studies in pediatric end-stage renal disease patients. The rich clinical and demographic information found in this database will facilitate study of important medical and non-medical risk factors for death, graft loss and cardiovascular disease among pediatric end-stage renal disease patients.

## Background

End-stage renal disease (ESRD) is rare in childhood. ESRD incidence rates in children range from 6.9 to 21.8 per million age related population in the 0-4 year old age group and 15-19 year old age group respectively [1]. As a result, individual pediatric nephrology centers have a limited number of patients receiving renal replacement therapy at any given time. Therefore, it is challenging for single centers to recruit sufficient numbers of patients to perform adequately powered clinical studies to evaluate treatment and outcomes in this patient population. More importantly, outcomes such as death and major cardiovascular events which require longitudinal follow-up over long periods of time are rarely documented within pediatric centers, because pediatric ESRD patients have usually been transferred to adult oriented care centers before these outcomes occur [2].

Multi-center longitudinal studies for evaluation of treatments and outcomes among pediatric ESRD patients are needed to overcome chronic challenges of inadequate power and lack of longitudinal follow-up. However, multi-center studies are costly, time consuming and not immediately feasible for many researchers in the field. Fortunately, there are several well established national and international registries documenting the course and outcomes of both adult and pediatric ESRD patients [3,4]. These include the United States Renal Data System (USRDS), [5] the United Network for Organ Sharing (UNOS),[6] the European Collaborative Transplant Study,[7] European Society of Pediatric Nephrology/European Renal Association-European Dialysis and Transplant Association, [8,9] and the Australia and New Zealand Dialysis and Transplant Registry [10]. Data from the Australia and New Zealand registry provided important information regarding long-term survival in pediatric ESRD patients [4]. There is also a well established pediatric dialysis and transplant registry, the North American Pediatric Renal Trials and Collaborative Studies (NAPRTCS) which has been the source of important pediatric outcomes data [11]. The most significant limitation of NAPRTCS is that patients have no further follow-up after age 21. In Canada, a well established organ failure registry (Canadian Organ Replacement Registry) has been collecting information on almost every single organ donation and transplant in Canada since 1981[1]. However, this registry has not been fully utilized to evaluate outcomes among Canadian pediatric ESRD patients.

Administrative databases used to collect data from Canada's single payer universal access healthcare system present a unique opportunity to study pediatric ESRD patients. Large national administrative health datasets document hospitalizations, outpatient encounters and emergency room visits for users of the health system [1]. Canadian hospital discharge data are routinely audited and the accuracy of data is greater than 95% [12]. Such databases have been used widely in Canada to develop surveillance programs and link processes to outcomes for various diseases [13-15]. Increasingly, administrative data are also being used to study diseases in childhood including asthma and inflammatory bowel disease [16-18].

This paper describes the creation of the Canadian Pediatric End-Stage Renal Disease database, a new database formed by combining administrative data from multiple sources to generate a large and rich, longitudinal dataset of Canadian pediatric ESRD patients. This new dataset will allow us to study long-term outcomes (death, allograft loss and major cardiovascular events) and risk factors for those outcomes in pediatric ESRD patients.

## Construction And Content

### Data sources

The Canadian Organ Replacement Register (CORR) is the sole national organ failure registry in Canada and is maintained by the Canadian Institute for Health Information [1]. All pediatric and adult dialysis and transplant centers in Canada submit data regularly to CORR using standardized paper forms. CORR data elements include demographics (age, date of birth, gender, personal health information number, ethnicity, postal code of residence), ESRD treatment modalities, change in treatment modalities with dates, treatment facility information, renal transplant details, selected comorbidities and outcomes (death, allograft loss). Height and weight at time of initiation of renal replacement are also recorded in the registry. Individual patient data is updated every 6 months.

The registry data have been used extensively in multiple clinical studies in patients with kidney failure [3,19-25]. A data quality study on the Canadian Organ Replacement Register was published in July 2009 [26]. This study involved a chart review for a sample of 1351 patient charts to assess documentation and coding practices at dialysis clinics, and the participation rate in CORR. In addition, CORR data were compared with data available in the national hospitalization database Canadian Institute for Health Information Discharge Abstract Database (CIHI DAD). It was determined that CORR captures 98.5% of renal transplants performed in Canada. Patient demographics were found to be coded with high reliability in CORR. In contrast, co-morbidities were recorded in CORR with low to moderate sensitivity, indicating a tendency to under-report co-morbidities to CORR. Nevertheless, co-morbidities were recorded with high specificity. The agreement rate between CORR data and chart review data on the primary renal disease code was 59%, indicating that primary renal disease could be more accurately reported to CORR.

The CIHI DAD is the national Canadian in-patient discharge abstract database containing demographic, clinical and institutional data [1]. Hospital discharge diagnoses are coded using International Statistical Classification of Diseases and Related Health Problems 9^th ^and 10^th ^revisions. In-hospital deaths and dates of death are also recorded. Each hospital discharge abstract record contains key demographic variables (date of birth, gender), personal health number assigned by relevant health authority, dates of admission, dates of discharge, up to 16 discharge diagnoses codes (most responsible diagnosis, pre- and post-admission co-morbidities) and up to 10 intervention codes. Special care unit length of stay is also documented. This dataset is available from the Canadian Institute for Health Information (CIHI) starting from fiscal year 1992 and data can be released to investigators with appropriate ethics approval and safeguards for privacy.

### Linked Data

Ethical approval was obtained from the University of Calgary to perform data linkages.

The CORR database was used to identify a cohort of incident pediatric ESRD patients in Canada except for the Province of Quebec (0-21 years) for the years 1992-2007. The Province of Quebec has separate privacy legislation and application for data release has been submitted.

Each patient identified in the registry was linked to their relevant hospital admissions in CIHI DAD using deterministic linkage methods. Deterministic linkage is accomplished by comparing identifying data fields data from two data sets leading to a judgment on whether two records refer to the same patient [27]. The data element used in the linkage algorithm for creation of this database consisted of only personal health card number and an exact match of health card number between two datasets was required to match a CORR registrant to corresponding hospitalization records in CIHI DAD [27]. Personal health information numbers are assigned by provincial health authorities and edit checks are performed during CORR data entry process. Personal health card numbers recorded in CORR are validated for length and alpha numeric characters and a check sum is computed for each health card number to detect accidental errors that may occur during data entry. CIHI DAD data has similar checking mechanisms to ensure the accuracy of health card numbers.

**Figure 1:**
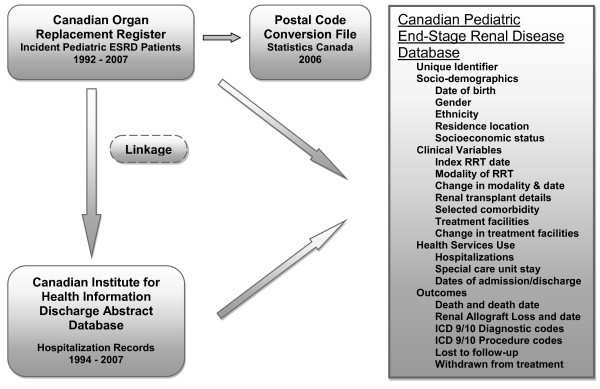
**Linkage of registry and administrative health data to create Canadian Pediatric End-Stage Renal Disease Database**. Abbreviations - RRT: renal replacement therapy; ICD 9/10: International Statistical Classification of Diseases (ICD-9-CM and ICD-10-CA)

Linkage was performed by personnel within CIHI to comply with privacy legislation. After linkage, each patient was assigned a unique identification number and the dataset was stripped of all personal identifying information. The merged database ***(Canadian Pediatric End-Stage Renal Disease Database) ***therefore comprises linked registry information and hospitalization records as shown in Figure 1. Selected variables and outcomes available in the database are shown in tables 1 and 2. The database is arranged longitudinally in time and includes key clinical information (index renal replacement therapy date, modality and changes in modalities of renal replacement, date of first transplant, clinical details of transplant, facilities of ESRD care, renal allograft loss dates and cause of renal allograft loss, hospitalizations, death date, cause of death).

**Table 1:** Variables available in Canadian Pediatric End-Stage Renal Disease Database

Sociodemographic variables
Gender

Date of Birth

Race

Region of Residence

Urban or Rural Residence

Neighborhood income quintile

Distance to nearest ESRD* care centers

**Selected Clinical variables**

Renal replacement therapy start date

First modality of renal replacement

Etiology of renal failure

Dates of change in renal replacement modality

Renal transplant dates

Donor type for transplant

*ESRD: end-stage renal disease

**Table 2:** Outcome variables in Canadian Pediatric End-Stage Renal Disease Database

Death and Cause of death
Lost to follow-up

Withdrawn from treatment (presumed death)

Renal Allograft loss and cause of allograft loss

Hospitalizations including special care unit admission

Major cardiovascular events

### Addition of Socioeconomic and Geographic Variables

The merged research database was supplemented with the addition of two key variables: a measure of socioeconomic status and geography for each patient. The six digit postal code for each study subject was obtained from CORR and linked to data from the 2006 Canadian Census using the Postal Code Conversion File (PCCF) to determine median neighborhood income quintile as one measure of socioeconomic status for each patient. PCCF changes with each census and socioeconomic variables are updated with time. The latest available PCCF was from year 2006. Residence location was defined as the distance between the residence of each patient and the nearest ESRD care centres. For these distance calculations, a comprehensive dictionary of postal codes for all Canadian ESRD care centres including satellite dialysis centres was created. Then, geographic coordinates for the center of the polygon defined by each 6 digit postal code (of patient residence and ESRD care centres) was determined using the PCCF and Canadian Postal Data Software (MelissaData, CA). These coordinates were then entered into ArcGIS 9 software (ESRI, CA) to determine the shortest distance by road between patient residence and nearest ESRD care centers. Using average posted speed, estimated travel times for individual patients to their nearest dialysis or transplant facilities were also calculated. Furthermore, we classified each patient residence location as urban or rural also using the PCCF available through Statistics Canada [28]. Investigators on this team have prior experience using these measures of socioeconomic status and residence location in studies involving the adult chronic kidney disease population [20,22,24,25].

## Utility

There were 1365 incident end-stage renal disease patients age ≤ 21 years (excluding province of Quebec) identified in the registry for the interval between 1992 and 2007, and 1200 (88%) health card numbers were available for linkage. Among these, 1011 (74.1%) were linked to CIHI DAD for at least one hospitalization episode. There were 9908 hospitalization records for the period 1994-2007 for matched patients (1992-1993 data was unavailable for linkage). We also performed an audit of death dates recorded in CORR using available in-hospital death dates. Of all deaths recorded in the database, 25% were in-hospital deaths, and were found in a hospitalization record. Among the in-hospital deaths, 95% of these were also found in CORR data with identical death dates for all except one date.

## Discussion

The research database described herein represents a major advance for clinical pediatric nephrology research. It is a rich source of information containing essential socio-demographic, clinical and health outcome variables that will facilitate longitudinal studies in a large and unselected cohort of pediatric ESRD patients. Beyond description of long-term patient and allograft outcomes, the future potential for such a research database includes assessment of medical risk factors, non-medical risk factors for various outcomes and quantification of health service utilization (in-patient only) in the entire cohort of Canadian pediatric ESRD patients. The research model can serve as a prototype for other countries with similar data availability, and provides an efficient and relatively inexpensive means of studying very long term outcomes in patients with an uncommon condition. The database may also facilitate international comparisons using similar data from other countries.

The single-payer, universal access health care system in Canada results in important advantages with respect to this type of database. This system ensures that all patients will have accessible administrative data files. This differs from the USRDS, in which administrative data are only available for patients covered under Medicare. Since transplant recipients currently lose Medicare eligibility after 3 years of graft function, administrative records are cut off three years after transplant for a significant proportion of transplant recipients in the USRDS [5]. The other major American database, the United Network for Organ Sharing, is a longitudinal database of American transplant recipients[6]. Although it contains information on virtually all transplant recipients in the US, contribution of follow-up data is not assured, because only UNOS member centers are obliged to submit data. Patients transferred to non-UNOS centers or to private physicians for long term transplant care will be lost to UNOS. Although UNOS data can be linked to USRDS administrative data, administrative records will only exist for patients covered under Medicare. These problems will not exist in the single-payer Canadian system.

Using routine demographic information collected, evaluation of potential disparities in access to kidney transplantation and time spent on dialysis among vulnerable sub-populations such as Aboriginal People will be immediately feasible with this dataset. The relative importance of socioeconomic status, which is a key determinant of health and wellbeing, can also be assessed for its contribution to patient and renal allograft outcomes and access to kidney transplantation. Equitable access to healthcare irrespective of ethnicity, gender and socioeconomic status is one of the central tenets of the Canadian universal access health care system. Therefore, the creation of this research database is a first step in examining 'at risk' pediatric populations for differences in kidney disease care received, and it may lead to identification of contributing factors and implementation of changes in health care delivery to optimize outcomes.

Geographical factors may play important roles in determining modality of renal replacement and patient outcomes among Canadian pediatric ESRD patients. To date, there are no studies examining the role of geography on pediatric patient outcomes. Canada has the second largest land mass for any country in the world and it has 11 pediatric ESRD care centers located only in major cities. Children living in rural remote areas need to travel long distances by road or air to their nearest pediatric ESRD care centre, and (since there may be no hemodialysis unit nearby) peritoneal dialysis may be the only option for those needing to start dialysis.

This approach will overcome some of the discontinuity in medical records that occurs when a pediatric patient is transferred to an adult center, effectively removing the boundaries on length of follow-up imposed by other registries such as the NAPRTCS; within the Canadian Pediatric End-Stage Renal Disease Database each patient can be followed until death if they reside in the country. This opens the door to studies examining outcomes occurring during the transition from pediatric to adult-oriented care - an interval considered to be at high risk for adverse outcomes [29,30]. For example, rates of renal allograft loss were examined during the transition period to adulthood using linked data from clinical records and administrative health datasets for a single center cohort of pediatric renal transplant patients from Ontario, Canada [31].

In addition to deaths, this database will capture major cardiovascular events (myocardial infarction, stroke, congestive heart failure) resulting in, or occurring during a hospitalization [32,33]. Cardiovascular end-points are usually not observed in childhood. A previous study linked data from medical charts to in-patient hospitalization records, and documented the incidence of major cardiovascular outcomes in a cohort of pediatric patients with end-stage renal disease [34]. However, this cohort was drawn from a single center and did not have access to full organ failure registry data - a limitation that the current dataset will overcome.

Although the potential for this research database is large, there are important limitations to consider. The use of registry and administrative data limits our ability to collect key clinical variables such as glomerular filtration rate and proteinuria which may influence patient and renal allograft outcomes. CORR data collection forms do not include these clinical variables; however, there is potential in the future to link to laboratory databases to obtain this information in certain jurisdictions. Registry data is submitted voluntarily by each center using paper based forms and potential for under-reporting exists. In terms of residence location, the postal codes are recorded on entry into CORR and we cannot capture residence moves presently. We are also unable to capture hospitalizations in other countries if patients travel abroad, although the likelihood of out-country hospitalizations occurring is negligible in this population. Determination of the cause of death from registry data is potentially problematic. Previous studies indicate that agreement between death certificates and data from dialysis registries is poor - perhaps because "kidney failure" is often listed as the cause of death on the former, or because many patients die at home (where cause of death cannot readily be ascertained) [35,36]. Currently, it is not feasible to obtain outpatient physician and emergency room encounters and therefore, the dataset is limited to inpatient encounters only. Finally, this dataset will not be able to capture the small proportion of deaths which occur at home unless they are reported in the registry.

Despite these limitations, this research database will result in a rich and useful data source. To maximize the potential of this dataset, ongoing updates of the dataset will be undertaken by adding incident patients each year and updating records for existing patients. At this stage, we have included data only for incident pediatric or young adult patients with ESRD (age ≤ 21) because this population is the focus for our research group; however, the linkage process could be easily replicated in the future for incident ESRD patients who are older than 21 years of age.

## Conclusions

Combining available national organ failure data with longitudinal hospitalization records can be a powerful tool facilitating clinical research among pediatric end-stage renal disease patients.

## Abbreviations

CIHI: Canadian Institute for Health Information; CIHI DAD: Canadian Institute for Health Information Discharge Abstract Database; CORR: Canadian Organ Replacement Register; ESRD: End-stage renal disease database; ESRI: Environmental Systems Research Institute; NAPRTCS: North American Pediatric Renal Trials and Collaborative Studies; PCCF: Postal Code Conversion File; USRDS: United States Renal Data System UNOS: United Network for Organ Sharing.

## Competing interests

The authors declare that they have no competing interests.

## Authors' contributions

SS, MT and BH conceived the idea for a linked database and led the team which created the database. SS wrote the manuscript and BF contributed sections of the manuscript. MT, BH and BF performed critical reviews of the manuscript. ANA and AS performed the data analysis and critically reviewed the manuscript. YN and RW facilitated and performed the creation of the linked database at the Canadian Organ Replacement Register and provided linkage method details for the manuscript. All authors read and approved the final manuscript.

## Pre-publication history

The pre-publication history for this paper can be accessed here:

http://www.biomedcentral.com/1471-2369/11/21/prepub
